# Chemical Markers in Italian Propolis: Chrysin, Galangin and CAPE as Indicators of Geographic Origin

**DOI:** 10.3390/plants13192734

**Published:** 2024-09-29

**Authors:** Elisabetta Miraldi, Giorgio Cappellucci, Giulia Baini, Elia Silvia Pistone, Marika Allodi, Gabriele Costantino, Chiara Spaggiari, Marco Biagi

**Affiliations:** 1 Department of Physical Sciences, Earth and Environment, University of Siena, Via Laterina, 8, 53100 Siena, Italy; elisabetta.miraldi@unisi.it (E.M.); giulia.baini2@unisi.it (G.B.); 2 SIFITLab, Italian Society of Phytotherapy, Via Laterina, 8, 53100 Siena, Italy; sifitlab@sifit.org (E.S.P.); 3 Department of Food and Drug, University of Parma (Department of Excellence 2023–2027), Parco Area delle Scienze, 43124 Parma, Italy; marika.allodi@unipr.it (M.A.); gabriele.costantino@unipr.it (G.C.); marco.biagi@unipr.it (M.B.)

**Keywords:** Italian propolis, flavonoids, pinocembrin, chrysin, CAPE, geographical diversity

## Abstract

Knowledge of the chemical composition of propolis is crucial for understanding the characteristics of products of different origins, but also for quality control and regulatory purposes. To date, official monographs or official analyses that allow researchers to evaluate propolis in a proper way have not yet been released. This study focuses on the characterization of twenty-seven Italian propolis samples and the identification of chemical markers that define its geographical provenance. Total polyphenol (TP) and total flavonoid (TF) content, alongside the quantification of pinocembrin, chrysin, galangin, and caffeic acid phenethyl ester (CAPE), were identified as potential markers. Additionally, DPPH assays were conducted to evaluate the antiradical activity of propolis samples. Our findings demonstrated that TPs, TFs and pinocembrin differed in propolis of different origins, especially in samples from the islands. However, the quantification of the sum of chrysin and galangin and CAPE provided a clearer distinction of the geographical origin of the propolis samples. In contrast, the DPPH assay did not prove useful for this purpose, as most results were similar and, therefore, not significant. This study lays the groundwork for future research on propolis. These findings could contribute to the development of more refined methods for distinguishing propolis origins, enhancing the understanding, valuation and quality control of this natural product in various applications.

## 1. Introduction

In relation to bee propolis, ethnopharmacology has reported and described similar properties for a variety of products. These products have been identified as having distinct botanical and geographic origins, but they share antioxidant, immunomodulatory, and antimicrobial (or more accurately, “anti-pathogen” activities) properties [1]. On the one hand, this recognizes the univocal role of propolis in worldwide beehives: a sanitizing putty, mainly made of leaf buds, but also of other plant secretions [2], consisting of volatile and non-volatile terpenes, wax and phenolic compounds [3]. On the other hand, these general concepts are not enough to move towards a rational application of bee propolis in the modern medicine. In-depth research of propolis pharmacology and chemistry is mandatory to investigate the mechanism of action, targets, and signaling involved in the activity of the specific constituents and particular phytocomplexes that characterize bee propolis of different origins. Given an example, artepillin C, pinocembrin and caffeic acid phenethyl ester (CAPE) are all phenolic compounds with an immunomodulatory effect, but artepillin C is a prenylated cinnamic acid found in green Brazilian propolis, known as an activator of TRPA1 channels [4], pinocembrin, an inhibitor of MAPK p38, is a flavanone common in Euro-Asian poplar propolis [5], while [5] CAPE, known as inhibitor of NF-κB, is a caffeic acid ester found in several propolis of different origin [6].

A higher knowledge of the chemical composition of propolis is crucial for understanding and maximizing the potential of different propolis.

Chemical analyses represent the most useful way to obtain information on the geographical provenance of propolis, but also for quality control purposes, a challenging concern not yet resolved. To date, there is no official Pharmacopoeia monograph available for bee propolis, nor any other official texts that include modern chemical assays [3,6]; currently, the best official reference is represented by the Chinese Pharmacopoeia, where a minimum of 2% of chrysin and 1% of galangin are required for raw propolis (poplar-type, mainly from *Populus* spp.) [7].

Moreover, the analysis and characterization of propolis and propolis-based formulations are also critical to regulatory issues. Indeed, the marketing and use of specific preparations of propolis, such as liquid ethanolic extracts, alcohol-free extracts or dry extracts, are allowed only depending on regulatory framework of different countries; to cite the example of European Union (EU), bee propolis is included only as mother tincture in some homeopathic Pharmacopoeias (such as in France and Germany) for the preparation of homeopathic medicines, and allowed as an ingredient for food supplements only if traditionally extracted with permitted solvents, without refining and purification, to not cross the frontier of novel foods [8]. Thus, the chemical characterization of a propolis-based product remains the best way to legitimatize or not the use of this bee product in various areas of human health. These concerns are seriously taken into account by the scientific community in Italy, being the country with the fastest growing market within the EU for human health products [9].

Italian propolis could be generically categorized as poplar-type propolis, although Gardini et al., [10], on the basis of chemical characteristics, proposed a distinction between propolis from the Mediterranean area and that from the temperate region, which includes the river Po plain area. To date, this study [10] could be considered the most important reference for Italian propolis for the number of samples tested (43 collected in 2013), and for the statistical analyses performed. In detail, the authors reported the quantification of total balsam content and more than 20 phenolic acids and flavonoids, showing that flavones, flavonols, flavanones and dihydroflavonols were higher in propolis from the Po plain area. In addition, the study showed that the chrysin content, the most enriched single flavonoid (as mean content) in samples divided by ecoregional provenance was higher in propolis from the Mediterranean and in the Po plain area compared to the content in propolis from other temperate areas located in the Northern and Central Regions or in Island Southern locations.

Finally, the study showed that, interestingly, some samples from Sicily and Sardinia were found to be poor in flavonoids and that a substantial difference in antioxidant activity was not recorded among the samples.

In a previous study conducted by Papotti et al. [11], 20 samples collected in 2007 in the area of Bologna (Emilia Romagna) were analyzed to compare different harvesting methods and solvents of extraction; as a result, in all samples, galangin, pinocembrin and pinobanksin 3-acetate were found to be the most enriched constituents, and the use of wooden wedge and acetone provided the highest yields in phenolics and the most impactful antioxidant activity.

A third paper [12] that considered five samples of Italian propolis [12] collected in 2007 in a different location of Central Italy focused mainly on allergenic esters benzyl cinnamate and benzyl salicylate and reported that chrysin and galangin were found to be the most important single constituents.

Considering these premises, in this work, we analyzed 25 samples of propolis collected in 2020 in different Italian geographical areas, according to the conventional division made by the Italian Ministry of Tourism (Northern, Central, Southern Regions) and two insular samples, specifically, one from Sicily and one from a Tuscan island (Isola di Capraia), were added. The aim of our work was to focus on Italian propolis to better investigate and to clarify the chemical composition of samples collected in different geographical areas, providing an update of and an insight into the current literature. Specifically, we aimed to develop methods for the identification of chemical markers in Italian propolis and to determine their geographic origin in order to ensure the quality of propolis product and to prevent misrepresentation and fraud in the propolis industry.

## 2. Results and Discussion

### 2.1. Chemical Analyses of Propolis Samples

#### 2.1.1. Total Polyphenol and Total Flavonoid Quantification

As reported in the reference of the Chinese Pharmacopoeia [7], but also in the cited literature [6,10,11] and again by Popova et al., 2007 [13], poplar propolis is mainly characterized by a high content of polyphenols and, in particular, of flavonoids; for this reason, the analysis of total polyphenols (TPs) and total flavonoids (TFs) represented the first analytical step of this work.

The analysis of TPs and TFs in propolis of different geographical Italian regions (Northern, Center, Southern, and Islands) revealed significant variations in the content of these bioactive compounds in different samples (Table 1).

Within propolis from the Northern Regions, the sample collected in Piedmont N1 (Biella province) markedly differed from other samples, showing low TP and TF content. Within the Central Regions, samples C13 from Latium showed marked differences from the other samples, revealing low TP and TF content, whereas C5 from the Florence countryside showed the highest TP content but medium TF content. In general, propolis from Tuscany, C1-C10 in this work, showed a very similar TP and TF content with respect to samples from Umbria, C11 and C12, probably due to the similar climatic conditions of these two regions. In propolis samples from Southern Regions, samples S4 and S5 from Apulia were those with the lowest content of TPs and TFs. In agreement with Gardini et al. [10], in propolis from Islands, we found the overall lowest content of TPs and in the case of I2 from Tuscany, also the lowest content of TFs.

Afterwards, in order to provide a more visual and comprehensive overview of the chemical similarities and differences across regions, the TP and TF content of the 27 samples, grouped by their geographical provenance, were summed. This approach allowed for a clearer and more understandable comparison of the results based on regional divisions, as reported in Table 2.

**Figure 1:**
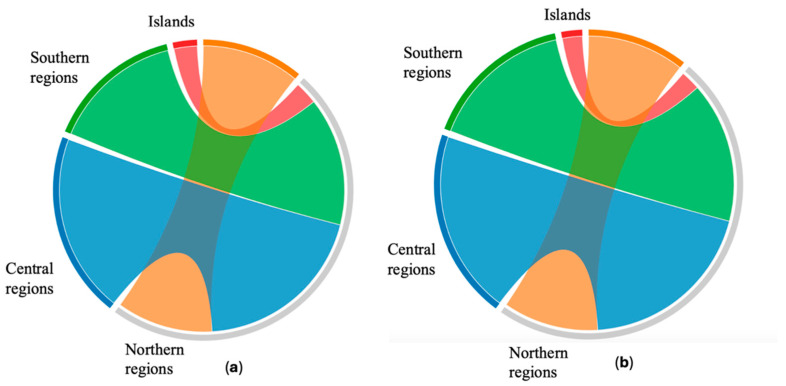
Chord diagrams representing the sum of (**a**) total polyphenols (TPs) and (**b**) total flavonoids (TFs) values divided into the four Italian geographical areas.

In propolis from Northern Regions, the mean TP content was 14.13 ± 6.47% *w*/*w*. This value was lower compared to that recorded for propolis from Central and Southern Regions but higher than that observed in samples from Islands (as graphically depicted in Figure 1, panel A). Propolis from Central Regions exhibited the highest mean TP content, 25.75 ± 5.41% *w*/*w*, revealing the same results for TFs, as depicted in Figure 1, panel A and B. Statistical analysis was performed to highlight the difference in the content of TPs and TFs within the four Italian regions. The results showed a significant difference in samples from Central Regions compared to samples from Northern Regions (*p* < 0.05), but not compared to samples from Southern Regions. In propolis from Southern Regions, the mean TP content was 18.85 ± 6.60% *w*/*w*, not statistically different from the mean value of propolis from Northern Regions.

Propolis samples from Tuscan and Sicilian islands displayed the lowest polyphenol content, with an average of 3.36 ± 0.45% *w*/*w*. Statistically significant differences were observed compared to samples from all other regions. Interestingly, despite clear distinct climatic conditions that characterize the Tuscan archipelago and Sicily, the two insular propolis samples maintained a similar poor content of TPs, shown in Figure 1 panel A, as already reported in [10] for propolis from Sicily and Sardinia.

As regards TFs (Table 2), these secondary metabolites composed the main phenolic subclasses in almost all samples, with the exception of I2; in many cases, TF represented more than 90% of TPs. The analysis of the ratio between TFs and TPs in samples divided by geographical area gave similar values, ranging from 0.63 ± 0.48 (Islands) to 0.83 ± 0.10% *w*/*w* (Southern Regions) (Table 2).

Propolis samples collected in the Northern Regions displayed a content of TFs of 10.82 ± 5.32% *w*/*w*. Similar to TP, the TF content in propolis from the Northern Regions (N) was lower than in propolis from the Central and Southern Regions but higher than in the Islands (Figure 1, panel A and B). Propolis samples from the Central Regions (C) also showed the highest mean TF content: 20.39 ± 3.65% *w*/*w*; the difference compared to the Northern Regions was statistically significant (*p* = 0.007), but the difference between propolis from Central and Southern Regions was not significant. The mean TF content in propolis from Southern Regions, in fact, was intermediate, 15.97 ± 6.09% *w*/*w*, with no significant difference found when compared to propolis from the Northern Regions. The two propolis samples collected in Italian islands deeply differed for TF content, even if both values were very low, 3.57 ± 0.12% *w*/*w* in propolis from Tuscan Capraia Island and only 0.90 ± 0.10% *w*/*w* in the Sicilian propolis. As in the case of TPs, differences were found compared to all other regions: Northern (*p* = 0.02), Central (*p* = 0.004), and Southern (*p* = 0.002).

In line with the aim of this work, the results obtained by analyzing propolis samples collected in different geographical Italian areas allowed us to deepen previous works and showed that results related to TPs and TFs content were in general in agreement with the work of Gardini et al. (2018) [10] and Popova et al. (2007) [13]. Nevertheless, we found that an insight could be made in order to distinct propolis from the Northern, Central, Southern Regions and Islands because of marked differences in TPs content, but also in TFs, as in the case of the comparison of Northern/Central and Islands with other areas.

Temperature and soil composition could be responsible for the variations in propolis composition, as well as the differing presence of *Populus* species and other plants in the Salicaceae family across various locations. However, regarding this latter point, it is important to note that in Italy, *Populus* species are primarily found in the regions of Piedmont, Lombardy, and Emilia-Romagna [14], where collected propolis samples were found to be poor both in TP and TF content (with the exception of N2). The question of the harvesting method, as claimed by Papotti et al. (2012) [11], newly emerges as a supplementary factor that could affect propolis quality.

#### 2.1.2. Quantification of Pinocembrin, Chrysin, Galangin and CAPE through HPLC-DAD

The UV methods used to quantify TPs and TFs have some important strengths because they are reliable, unexpensive and rapid, and therefore very useful for a preliminary chemical screening of multiple samples. Nevertheless, these methods are not sufficient to provide a high-quality characterization of natural products and, in the case of propolis, they do not allow us to investigate more subtle quali–quantitative differences in the flavonoid profile of samples from different Italian areas.

**Figure 2:**
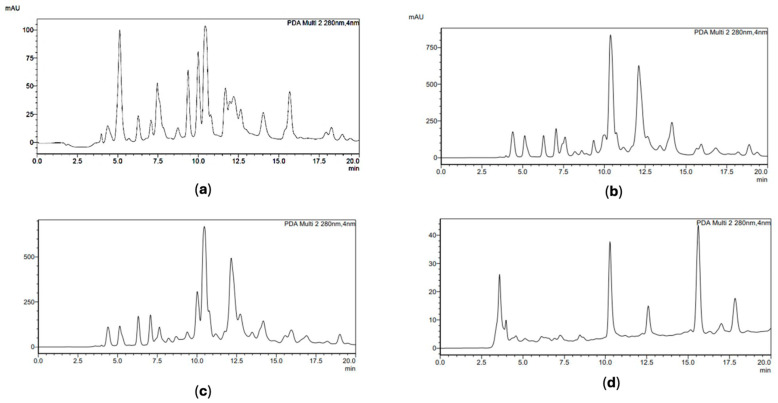
Chromatograms recorded at 280 nm. (**a**) Sample N1 from Piedmont (Biella); (**b**) sample C11 from Umbria, Perugia province; (**c**) sample S6, Calabrian propolis from Cosenza province; (**d**) sample I2 from Sicily (Messina). Pinocembrin (PIN) retention time (RT) = 10.4 min, CAPE at RT = 11.2 min, chrysin (CHR) at RT = 12.1 min, galangin (GAL) at RT = 12.5 min.

HPLC-DAD analysis was therefore performed to characterize the flavonoid fraction of propolis under analysis and allowed for the identification of the main constituents. In accordance with what was previously reported by Biagi et al. (2016) [9], we identified pinocembrin (PIN), with a retention time (RT) of 10.4 min. However, for chrysin (CHR) and galangin (GAL), we were not able to perfectly separate these two peaks that partially overlapped with an RT of 12.1 and 12.5 min., respectively. Therefore, we chose to quantify CHR and GAL together to avoid errors depending on different overlays recorded in samples. Caffeic acid phenethyl ester (CAPE) was identified and quantified in all samples at RT = 11.2 min. Figure 2a–d shows the chromatogram of N1, C11, S6 and I2, representative of samples from different origins.

PIN was found to be the flavonoid with highest content in several samples, as reported in Table 3. In particular, in five samples from Central Regions, its content was >10% *w*/*w*. In propolis from Northern Regions, in two (N2, N5) out of five samples, PIN ranged from 5.5% *m*/*m* to 8.2% *w*/*w*. With the exception of S4, in propolis from the Southern Regions, PIN ranged from 5.1% *w*/*w* to 9.9% *w*/*w*. Finally, in samples from the Islands, PIN was found in high amount compared to TFs.

Similarly to what was previously carried out with regard to the TPs and TFs in Table 2, in order to provide a general overview of the chemical differences within the four Italian geographical areas of interest, a quantification of the identified flavonoids and CAPE was carried out, and the ratios of PIN and TFs, CHR and GAL and TFs, CAPE and TPs were calculated, as reported in Table 4.

The mean content of PIN in propolis from the Northern Regions was 4.26 ± 3.24% *w*/*w*, that from the Central Regions was 8.80 ± 2.18% *w*/*w*, that from the Southern Regions was 7.10 ± 2.28% *w*/*w*, and in the Islands, it was 0.85 ± 0.86% *w*/*w*. The statistical analysis revealed a significant difference in PIN content by comparing the Northern and Central Regions (*p* = 0.003), and in insular propolis compared to all other samples. PIN content was found to be correlated to TFs and the ratio between PIN and TFs was found to have similar values for propolis from all geographical areas. In conclusion, with regard to PIN content, as already suggested by Gardini et al. (2018) [10] but also by Cui-Ping et al. (2015) [15] for Chinese propolis, in this work, we reinforced the opinion that PIN could be considered a general marker of poplar-type propolis, but we also noticed that its high content distinguished propolis from the Central and Southern Regions from that of the Northern Regions and Islands.

Regarding CHR and GAL, a larger variation was recorded. In propolis from the Northern Regions, the CHR and GAL content ranged from <0.05% (N4) to 4.87% *w*/*w* (N5)**.** In propolis from the Central Regions, the range was 3.15% (C13) up to 10.03% *w*/*w* (C11)**,** and in propolis from the Southern Regions, the values ranged from 1.20% (S4) up to 12.22% *w*/*w* (S1). The Islands (I) presented the lowest CHR and GAL content (0.28% and 0.07% in I1 and I2, respectively), reinforcing the distinctiveness of insular propolis compared to mainland samples.

The very low CHR and GAL content obtained with the sample from the Alps, in Trentino, the one collected at the highest altitude (>500 m), was considered a point worthy of further investigation, which is currently difficult to discuss without other references from the same source.

The mean content of CHR + GAL in samples collected in the Northern Regions was 2.16 ± 1.93% *w*/*w*, a value statistically different compared to the content of CHR + GAL in samples from the Central Regions (6.73 ± 2.22% *w*/*w*, *p* = 0.002), but also compared to values recorded in samples from the Southern Regions (6.01 ± 3.66% *w*/*w*, *p* = 0.04). On the other hand, the mean content of CHR + GAL in samples from the Central and Southern Regions did not differ in a significant manner. Propolis from the Tuscan archipelago and Sicily once again deeply differed from the others from the Central and Southern Regions (and Northern, even if not in statistically significant manner). A less marked difference was noted in the ratio between CHR + GAL and TF content among samples from the north, center and south, but, similar to PIN, the statistical analysis showed a significant difference between propolis from the north and south (*p* = 0.04). Again, a clear difference was observed by comparing propolis from Islands and those from the Central and Southern Regions (*p* < 0.001) (Table 4). 

In this study, we also analyzed the content of CAPE in all samples, being one of the most peculiar caffeic acid derivatives of propolis [16] but rarely taken into account in large comparative analyses.

As showed in Table 3, the range of CAPE content was narrower than in the other parameters previously considered, and in 22 of the 27 samples, CAPE ranged between 1.0% and 2.0% *w*/*w*. Nevertheless, in line with previous findings here obtained, in three out of five samples from the Northern Regions (N1, N3, N4), CAPE was below 1% *w*/*w* and a very low amount of this compound was also found in insular samples. As summarized in Table 4, samples from the Northern Regions had a moderate CAPE content (1.06 ± 0.26% *w*/*w*), and propolis from the Central Regions had a mean content of 1.51 ± 0.27% *w*/*w*, whereas samples from Southern Regions had the highest mean content of CAPE: 1.62 ± 0.28% *w*/*w*; in line with our previous findings, insular regions presented the lowest CAPE content (0.12 ± 0.03% *w*/*w*). The statistical analysis revealed that CAPE content in propolis from the Northern Regions was significantly different from that of the Central and Southern Regions (*p* = 0.01 and *p* = 0.006, respectively) and, as observed for other parameters, a great difference was recorded comparing CAPE content in propolis from the Islands and that of all other samples (*p* < 0.001 for all comparisons).

A differential study of CAPE content with respect to TPs was finally performed (Table 4) and low differences in samples from the Northern, Central and Southern Regions were observed, but a lower ratio was found in propolis from Islands, with differences statistically significant between samples from the Northern and Southern Regions (*p* = 0.03 and *p* = 0.006, respectively).

**Figure 3:**
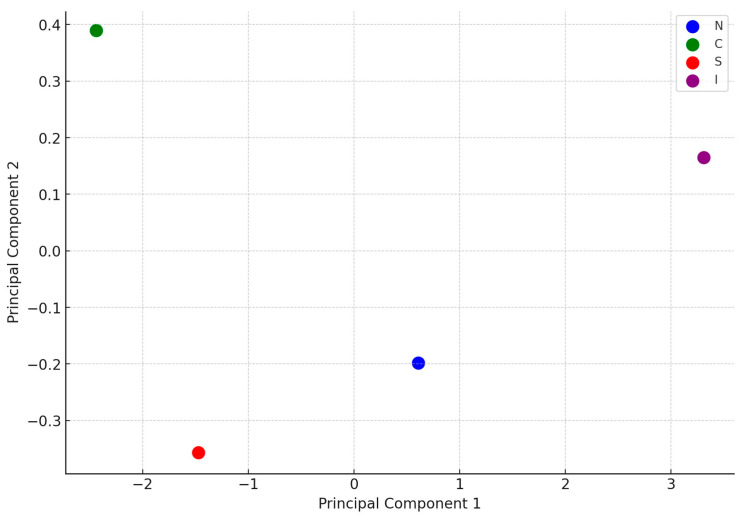
Principal component analysis (PCA) plot built (Phyton) with % *w*/*w* of pinocembirn, chrysin and galangin, CAPE, total polyphenols and total flavonoids grouped by geographical areas.

The principal component analysis (PCA) plot (Figure 3) visually represents the differences in the chemical composition of propolis samples from various geographical regions. Each region is depicted using different colors, with the Northern Region shown in blue, the Central Region in green, the Southern Region in red, and the Islands in purple. The separation of these regions on the plot indicated that the chemical markers used in the analysis (such as PIN, CHR, GAL, and CAPE, TPs and TFs) effectively distinguished the samples based on their geographical origin. The insular samples were notably distinct from the others, appearing clearly separated on the plot, which suggested a different chemical profile for these samples. This distinctiveness could be attributed to the specific environmental conditions or plant sources found on the islands. The Northern and Central samples, while closer to each other, still showed noticeable differences, implying some level of similarity between them, yet with distinct characteristics. The southern samples also formed a separate group, highlighting their unique chemical profile compared to those of the Northern and Central Regions. Overall, the PCA analysis provided a clear and effective visualization of how geographical factors influence the chemical composition of propolis. The distinct clustering of samples from different regions supported the conclusion that these regions have unique propolis profiles, particularly emphasizing the uniqueness of the insular samples. This visualization strengthened the understanding of regional variations in propolis composition. In this work, we could update and simplify the division of geographical clusters of Italian propolis, emphasizing a marked difference in propolis from the Northern Regions with respect to those from the Central and Southern Regions, more enriched in TPs and TFs and PIN, but also, and more specifically, in CHR + GAL and CAPE. However, the most evident difference in propolis samples was observed for those collected in Sicily and in Capraia, which were chemically very poor. Partly not in accordance with the work of Gardini et al. [10], we could not distinct in a clear way propolis collected near the seacoast and far from the sea and, more in general, we observed strong similarities in propolis from the Central and Southern Regions, regardless of latitude and altitude. Differently from Gardini et al. [10], unfortunately, we were not able to collect and analyze propolis from Po plain areas and we consider this a limitation of this work that did not allow us to provide a supplementary comparison. Moreover, as mentioned before, the very low content of CHR + GAL in the sample collected above 500 m in the Alps led to interest in focusing on mountain propolis in the future to understand if the different flavonoid profile may be a peculiar characteristic.

### 2.2. Antiradical Activity of Propolis Samples

In line with other studies [17,18], we also performed a simple, but validated antiradical assay through the DPPH (2,2-diphenyl-1-picrylhydrazyl) test.

Of note, we measured a strong activity for most of the samples, showing activity ≤ 100 μg/mL for all samples with the exception of insular propolis. Samples from the Central Regions showed the narrowest range of IC_50_ values, whereas samples from the Southern Regions showed the largest range of activity (Table 5).

**Figure 4:**
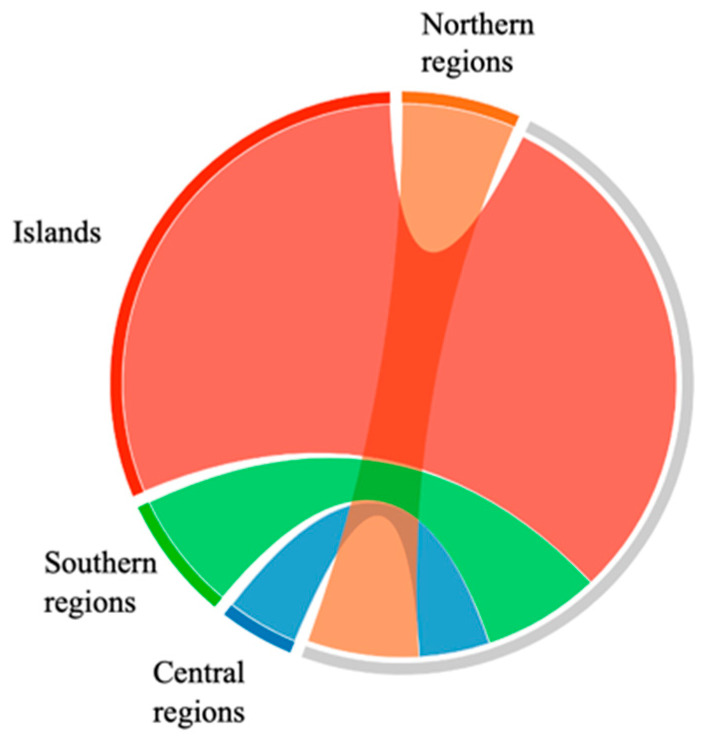
IC_50_ in DPPH test of propolis samples divided by geographical origin.

The analysis of the grouped samples, divided by geographical areas, showed similar mean IC_50_ values for samples from the north, center and south (41.33 ± 15.36, 26.46 ± 4.09 and 43.68 ± 28.60 μg/mL, respectively), but a much higher mean value for insular samples, as depicted in Figure 4. (*p* < 0.001 for all comparisons) (Table 6 and Figure 4).

Delving into a possible correlation between antiradical activity and TPs, PIN and CAPE content through IC_50_ analysis of individual samples, an R^2^ between 0.52 and 0.54 could be observed. However, the most valid correlation was found between the IC_50_ values and TFs content (R^2^ = 0.59), and within the samples, the correlation was higher between those from the north, south and islands.

On the other hand, the correlation between the antiradical activity and CHR + GAL content was the worst (R^2^ = 0.37), which could be an explanation of the limited effect of geographical diversity of the tested samples in the DPPH test. In fact, we postulated that scarce differences observed in the assay mainly depended on the high content of red/ox active species present in all samples and we could say that, in propolis, the whole phytocomplex played a major role in antiradical and antioxidant activities. In addition, our findings regarding the similar IC_50_ values in DPPH test of propolis collected in different places are again in agreement with Gardini et al. [10], who also reported similar scavenging/antioxidant activities of samples obtained through other assays. A future perspective about this point is to study fine red/ox activity differences in different propolis exploiting the sensitivity and specificity of purely chemistry-based methods such as voltammetry, which has already been demonstrated to be effective and reliable for this bee product [19]. Interestingly, after having discussed the scarce utility of the DPPH test, we confirmed that, as regards propolis, chemical insights as those determined in this work are crucial to discriminate different samples, in this case, from different Italian areas.

Overall, our study aligns with that of Kasote et al. (2022) [20], who emphasized marked differences in propolis across world regions, alongside local- and region-specific uniqueness in chemical composition.

Expanding to global propolis, chemical analyses can distinguish different types, often linking them to their botanical sources. For example, Brazilian green propolis, the second most studied type for health purposes (after poplar-type), is rich in artepillin C and caffeic acid derivatives, unlike poplar propolis, which contains more flavonoids. This difference stems from its botanical source, *Baccharis* spp. [21]

A study by Blicharska and Saidel (2019) [22] clarified that African propolis exhibits diverse phytochemical profiles due to its varied ecosystems. Notably, Cameroonian propolis was found to be rich in terpenoids, Kenyan propolis in unique xanthone derivatives, whereas in Algerian propolis, flavonoids were found in poplar-type propolis, such as pinobanksin, PIN, GAL, and CHR, were identified.

Our study delved deeper into the regional differences in Italian propolis and a comparison with others from different Mediterranean regions revealed interesting similarities and differences.

In Greece, for example, Kalogeropoulos et al. (2009) [23] and Kasiotis et al. (2017) [24] found that Greek propolis collected in different parts, including Corfu, Creta and Kos Island and Arkadia, Peloponnese, Argolis and Central Macedonia, showed a similar qualitative flavonoids profile, consisting of pinobanksin 3-acetate, apigenin and PIN, CHR and GAL, but specific quantitative patterns, which is consistent with the findings of our study. They also reported high antioxidant activity of Greek propolis, attributed to these compounds.

A study by Kumazawa et al. (2013) [25] analyzed the chemical composition of 28 Andalusian propolis and, despite some differences in individual samples, identified pinobanksin, pinobanksin 3-acetate, PIN, CHR and GAL as the most abundant flavonoids, but also significant amounts of caffeic acid derivatives, including CAPE, one of the key compounds identified in our study, especially in samples from the Central and Southern Regions. This supports the notion that Mediterranean propolis, particularly in southern regions, shares common characteristics in its chemical composition, which could be related to the similar environmental and botanical sources available in these areas.

A study on 56 Croatian propolis samples published by Saftić et al. (2019) [26] also emphasized the clear chemical diversity in samples collected from continental and coastal areas. Although the study did not analyze the most abundant flavonoids in detail, a targeted and untargeted phenolic profiling suggested a division between typical poplar-type propolis (from continental Croatia) and Mediterranean propolis (from the coastal area). In fact, in the study, it was noted that propolis samples from the Adriatic coast exhibited a distinct chemical profile with a higher ratio between diterpenes and flavonoids; these findings are in line with our observation of regional differences in Italy, particularly the distinct composition of propolis from island regions like Capraia and Sicily, which are more likely attributable to Mediterranean-type propolis

## 3. Conclusions

Chemical analyses of different propolis are pivotal for the rational and modern use of this fascinating bee product for human health purposes, as well as being the most important means of characterizing products from different geographical areas.

In this study, we aimed to update the knowledge of the chemical features of Italian poplar-type propolis by analyzing 27 samples collected in the same year from various regions covering Northern, Central, and Southern Italy, as well as Capraia Island in the Tuscan archipelago and Sicily.

Our findings revealed that it is possible to effectively distinguish propolis from different geographical regions using straightforward and rapid UV methods, such as total polyphenol and total flavonoid analyses. Additionally, the quantification of key markers like pinocembrin, chrysin, galangin, and CAPE via HPLC-DAD enabled a more precise determination of the geographical origin of propolis. While the DPPH assay provided valuable insights into antiradical activity, it proved suboptimal for differentiating geographical sources due to the similarity in the results.

The main strength of this work lies in having established a solid foundation for developing more refined methods to assess and ensure the quality and origin of propolis.

We are fully aware that this study represents only a preliminary basis that needs further development. This includes expanding the comparison to include sub-areas (such as fluvial regions, Apennine and Alpine propolis, and samples from inland beehives in Sicily and Sardinia) and significantly improving the analytical level beyond the analysis of the main constituents.

## 4. Materials and Methods

### 4.1. Sample Collection

Twenty-seven propolis samples were obtained from beekeepers in different regions throughout the Italian peninsula and islands. All propolis were produced during spring 2020 and the samples were supplied already cleaned and de-waxed. In the laboratory, they were solubilized in EtOH 75% (*v*/*v*) at a concentration of 10 mg/mL. The solutions were all yellow-orange in color, tending to red-brown. The odor was balsamic.

Details of areas where the samples were collected are shown in Table 7.

### 4.2. Phytochemical Analyses

#### 4.2.1. Quantification of Total Polyphenols and Total Flavonoids

The total polyphenol content was quantified by the Folin–Ciocâlteu (FC) colorimetric assay according to Finetti et al., 2020 [27] using ethanolic solution of propolis samples 10 mg/mL. The absorbance of each solution was measured with a spectrophotometer (UV/Vis spectrophotometer UV-1900i, Shimadzu, Kyoto, Japan) at 700 nm, using as a reference a blank consisting of ethanol reacted under the same conditions. The calibration line was made with gallic acid at concentrations between 0.06 and 5 mg/mL, with an R^2^ > 0.99. The concentration of polyphenols in the samples was calculated as % *w*/*w*, expressed as gallic acid equivalent (GAE).

Total flavonoids were quantified according to the method reported by Governa et al., 2020 [28] and Sberna et al., 2022 [29]. tHE Samples (10 mg/mL in ethanol) were diluted 1:200 in ethanol 75% *v*/*v* and, using a VICTOR Nivo 3S multi-mode plate reader, (PerkinElmer, Waltham, MA, USA), absorbance was read at 353 nm, and galangin was used as a standard (0.05–2 mg/mL, R^2^ > 0.98).

All tests were conducted in triplicate.

#### 4.2.2. HPLC-DAD Analysis

HPLC analyses were conducted using a Shimadzu Prominence LC 2030 3D instrument equipped with a Bondapak^®^ RP C18 column, 10 μm, 125 Å, 3.9 mm × 300 mm (Waters Corporation, Milford, MA, USA). The solvents used were as follows: A: water + 0.1% formic acid; B: methanol + 0.1% formic acid (Merck Sima-Aldrich, Darmstadt, Germany).

The chromatographic conditions were as follows: B from 60% at 0.01 min to 70% at 6.00 min and to 85% at 17.00 min and 3 min for returning to the initial conditions. The total run time was 20 min. Flux was set at 0.75 mL/min. Chromatograms were recorded at 280 nm.

Pinocembrin, chrysin, galangin and CAPE of reference standard grade (Merck Sima-Aldrich) were used. The method guaranteed linearity and precision (R^2^ > 0.99 for all standards), repeatability (inter- and intra-day differences in replicates < 15%) and allowed us to quantify all metabolites of interest in tested samples above the limit of quantification (LOQ), <0.05 μg in column.

The samples (10 mg/mL in ethanol) were diluted 10-fold in ethanol, filtered 0.44 μm and injected (10 μL).

Compounds peaks were identified by comparing their retention times and UV spectra with those of the corresponding standards.

The analyses were conducted in triplicate.

### 4.3. Antiradical Activity of Propolis

The antiradical activity of propolis was determined using the DPPH (2,2-diphenyl-1-picrylhydrazyl) as described by Bonetti et al., 2021 [17]. The negative control was made with ethanol and DPPH (1:19). Pure ascorbic acid (Merck Sigma-Aldrich) was used as the reference substance. The percentage inhibition of DPPH was calculated according to the following formula, % inhibition = (Absc − Absx)/Absc × 100, and IC_50_ was calculated.

### 4.4. Multivariate Modeling

Principal component analysis (PCA) was performed to assess the variation in propolis composition across different geographical regions. The data used for this analysis included the concentrations of key compounds such as pinocembrin, chrysin + galangin, CAPE, total polyphenols and total flavonoids from propolis samples, specifically, the average of the four geographical regions: Northern (N), Central (C), Southern (S), and Islands (I). The data were centered and scaled to ensure comparability between the variables. PCA was conducted using Python programming language, specifically with the scikit-learn library [30] for PCA computation and the matplotlib library for visualization [31]. The analysis used a covariance matrix to identify the principal components that explain the maximum variance in the dataset. The first two principal components, which accounted for the most variance, were plotted to visualize the clustering and distribution of the samples from each region. Each region is represented by a distinct color on the scatter plot.

### 4.5. Statistical Analysis

Data were presented as mean ± standard deviation (SD) of experimental triplicates. Statistical analyses were performed using one-way analysis of variance (ANOVA) followed by post hoc Tukey’s test (with *p* < 0.05 as significance level).

## Figures and Tables

**Table 1:** Content of total polyphenols, expressed as gallic acid equivalent, and total flavonoids, expressed as galangin equivalent. Sample are grouped according to different geographical Italian areas, Northern (samples N1–N5), Central (C1–C13), Southern Regions (S1–S7) and Islands, namely, Capraia Island (Tuscany), I1 and Sicily, I2. Values are expressed as a percentage *w*/*w* (mean ± SD).

Sample Code	TPs (% *w*/*w*)	TFs (% *w*/*w*)	Sample Code	TPs (% *w*/*w*)	TFs (% *w*/*w*)
**N1**	5.34 ± 0.64	3.38 ± 0.17	**C10**	23.84 ± 1.08	21.78 ± 1.78
**N2**	21.86 ± 3.07	18.13 ± 0.06	**C11**	22.70 ± 2.06	19.63 ± 2.36
**N3**	10.45 ± 1.88	9.26 ± 0.18	**C12**	27.36 ± 3.35	21.42 ± 0.98
**N4**	18.20 ± 2.09	11.04 ± 0.37	**C13**	16.58 ± 1.61	11.60 ± 0.14
**N5**	14.78 ± 3.28	12.28 ± 0.63	**S1**	27.88 ± 4.24	24.28 ± 3.27
**C1**	28.31 ± 4.61	26.64 ± 2.01	**S2**	21.41 ± 2.58	16.65 ± 0.05
**C2**	23.24 ± 4.74	21.29 ± 0.02	**S3**	22.53 ± 2.06	19.90 ± 0.65
**C3**	22.67 ± 4.28	18.15 ± 0.60	**S4**	8.26 ± 2.19	5.33 ± 0.01
**C4**	27.85 ± 6.44	20.70 ± 2.16	**S5**	12.10 ± 1.04	11.39 ± 0.01
**C5**	36.41 ± 0.30	18.33 ± 2.29	**S6**	20.14 ± 2.30	16.56 ± 0.90
**C6**	31.45 ± 4.82	25.65 ± 3.74	**S7**	19.64 ± 0.22	17.67 ± 0.04
**C7**	31.90 ± 5.78	20.50 ± 1.45	**I1**	3.68 ± 0.37	3.57 ± 0.12
**C8**	21.67 ± 2.30	18.94 ± 0.24	**I2**	3.04 ± 0.15	0.90 ± 0.10
**C9**	20.82 ± 2.01	20.43 ± 0.06			

**Table 2:** Total polyphenol content expressed as gallic acid equivalent, and total flavonoids, expressed as galangin equivalent. Samples are grouped according to geographical areas: Northern (N)), Central (C), Southern Regions (S) and Islands (I), Values are expressed as a percentage *w*/*w* ± SD (mean ± standard deviation). Ratio between TFs and TPs in propolis divided by geographical area, N, C, S and I, is also reported. Different letters indicate significant differences among values for grouped samples (*p* < 0.05 according to one-way analysis of variance, ANOVA, followed by post hoc Tukey’s test).

Geographical Region	TPs (% *w*/*w*)	TFs (% *w*/*w*)	Flavonoid/Polyphenol Ratio
**N**	14.13 ± 6.47 ^a^	10.82 ± 5.32 ^a^	0.76 ± 0.13 ^a^
**C**	25.75 ± 5.41 ^b^	20.39 ± 3.65 ^b^	0.81 ± 0.13 ^a^
**S**	18.85 ± 6.60 ^ab^	15.97 ± 6.09 ^ab^	0.83 ± 0.10 ^a^
**I**	3.36 ± 0.45 ^c^	2.24 ± 1.89 ^c^	0.63 ± 0.48 ^a^

**Table 3:** Content of pinocembrin (PIN), the sum of chrysin and galangin (CHR + GAL) and caffeic acid phenethyl ester (CAPE) in different propolis samples expressed as a percentage *w*/*w* (mean ± SD).

Sample Code	PIN (% *w*/*w*)	CHR and GAL (% *w*/*w*)	CAPE (% *w*/*w*)	Samples Code	PIN (% *w*/*w*)	CHR and GAL (% *w*/*w*)	CAPE (% *w*/*w*)
**N1**	1.04 ± 0.14	0.60 ± 0.01	0.86 ± 0.41	**C10**	10.16 ± 0.05	8.67 ± 0.14	1.65 ± 0.05
**N2**	8.20 ± 0.16	2.79 ± 0.17	1.30 ± 0.04	**C11**	9.30 ± 0.08	10.03 ± 0.34	1.70 ±0.04
**N3**	2.28 ± 0.10	2.52 ± 0.07	0.96 ± 0.03	**C12**	11.27 ± 0.05	7.13 ± 0.13	1.62 ± 0.07
**N4**	* coeluition	<0.05	0.81 ± 0.03	**C13**	4.91 ± 0.05	3.15 ± 0.07	1.01 ± 0.05
**N5**	5.53± 0.03	4.87 ± 0.11	1.37 ± 0.01	**S1**	9.87 ± 0.32	12.22 ± 0.13	1.93 ± 0.05
**C1**	10.07 ± 0.12	9.38 ± 0.15	1.52 ± 0.02	**S2**	8.14 ± 0.15	4.55 ± 0.01	1.96 ± 0.05
**C2**	8.16 ± 0.15	5.68 ± 0.10	1.80 ± 0.02	**S3**	8.33 ± 0.03	6.24 ± 0.10	1.43 ± 0.01
**C3**	6.88 ± 0.09	5.38 ± 0.01	1.16 ± 0.01	**S4**	3.08 ± 0.01	1.20 ± 0.03	1.59 ± 0.03
**C4**	8.05 ± 0.09	6.11 ± 0.18	1.53 ± 0.01	**S5**	5.13 ± 0.22	2.63 ± 0.12	1.54 ± 0.05
**C5**	8.01 ± 0.24	6.58 ± 0.42	1.37 ± 0.01	**S6**	8.01 ± 0.84	7.53 ± 0.42	1.74 ± 0.21
**C6**	11.53 ± 0.12	10.02 ± 0.40	1.57 ± 0.01	**S7**	7.13 ± 0.13	7.70 ± 0.14	1.17 ± 0.07
**C7**	5.14 ± 0.17	4.31 ± 0.18	1.19 ± 0.01	**I1**	1.46 ± 0.10	0.28 ± 0.01	0.14 ± 0.03
**C8**	9.97 ± 0.03	4.57 ± 0.04	1.54 ± 0.43	**I2**	0.24 ± 0.11	0.07 ± 0.01	0.10 ± 0.01
**C9**	10.95 ± 0.13	6.47 ± 0.27	1.96 ± 0.41				

* Quantification vitiated by a marked overlay: approximate value ranging from 4.5% to 6.0%.

**Table 4:** Content of pinocembrin (PIN), the sum of chrysin and galangin (CHR + GAL) and CAPE and relative total flavonoid (TF) and total polyphenol (TP) ratio found in samples from Northern (N)), Central (C), Southern Regions (S) and Islands (I). Values are expressed as a percentage *w*/*w* (mean ± SD). Different letters indicate values significantly different (*p* < 0.05), according to ANOVA followed by Tukey’s post hoc test.

Geographical Region	PIN (% *w*/*w*)	PIN/TF Ratio	CHR and GAL (% *w*/*w*)	CHR and GAL/TF Ratio	CAPE (% *w*/*w*)	CAPE/TP Ratio
N	4.26 ± 3.24 ^a^	0.29 ± 0.19 ^a^	2.16 ± 1.93 ^a^	0.20 ± 0.15 ^a^	1.06 ± 0.26 ^a^	0.09 ± 0.04 ^a^
C	8.80 ± 2.18 ^b^	0.43 ± 0.08 ^a^	6.73 ± 2.22 ^b^	0.33 ± 0.08 ^ab^	1.51 ± 0.27 ^b^	0.06 ± 0.02 ^ab^
S	7.10 ± 2.28 ^ab^	0.46 ± 0.06 ^a^	6.01 ± 3.66 ^b^	0.35 ± 0.11 ^b^	1.62 ± 0.28 ^b^	0.10 ± 0.05 ^a^
I	0.85 ± 0.86 ^c^	0.34 ± 0.10 ^a^	0.18 ± 0.15 ^c^	0.08 ± 0.01 ^ca^	0.12 ± 0.03 ^c^	0.04 ± 0.01 ^ab^

**Table 5:** IC_50_ of samples in DPPH assay, expressed as µg/mL. The standard deviation for all measures is <20% of the mean calculated value.

Samples	IC_50_ (µg/mL)	Samples	IC_50_ (µg/mL)
**N1**	67.27	**C10**	23.67
**N2**	27.34	**C11**	26.78
**N3**	40.76	**C12**	18.92
**N4**	37.78	**C13**	32.42
**N5**	33.48	**S1**	21.93
**C1**	25.82	**S2**	32.71
**C2**	30.04	**S3**	29.48
**C3**	26.80	**S4**	100.16
**C4**	27.09	**S5**	64.75
**C5**	24.12	**S6**	26.24
**C6**	27.70	**S7**	30.46
**C7**	26.18	**I1**	162.67
**C8**	36.13	**I2**	220.59
**C9**	24.25		

**Table 6:** IC_50_ in DPPH test of propolis samples divided by geographical origin. Different letters indicate values significantly different (*p* < 0.05), according to ANOVA followed by Tukey’s post hoc test.

Geographical Region	IC_50_
**N**	41.33 ± 15.36 ^a^
**C**	26.46 ± 4.09 ^a^
**S**	43.68 ± 28.60 ^a^
**I**	191.63 ± 40.96 ^b^

**Table 7:** List of analyzed samples with their origin. The labels on the samples indicate geographical areas of collection: N: Northern, C: Central, S: Southern, I: Islands.

Samples Code	Area of Origin	Gps Coordinates	Region	Samples Code	Area of Origin	Gps Coordinates	Region
**N1**	Valdilana (BI)	45°39′25.66″ N 8°09′01.85″ E	Piedmont	**C10**	Arcidosso (GR)	42°52′20″ N 11°32′15″ E	Tuscany
**N2**	Arcisate (VA)	45°51′18.98″ N 8°52′03.3″ E	Lombardia	**C11**	Castello delle Forme, Marsciano (PG)	42°58′47.06″ N 12°21′22.21″ E	Umbria
**N3**	Castellanza (VA)	45°37′ N 8°54′ E	Lombardia	**C12**	Deruta (PG)	42°59′ N 12°25′ E	Umbria
**N4**	Pergine Valsugana (TN)	46°04′ N 11°14′ E	Trentino-Alto Adige	**C13**	Norma, Monti Lepini (LT)	41°35′ N 12°58′ E	Lazio
**N5**	Castel San Pietro Terme (BO)	44°23′52″ N 11°35′22″ E	Emilia-Romagna	**S1**	Bellante (TE)	42°45′ N 13°48′ E	Abruzzo
**C1**	Quarrata (PT)	43°50′51″ N 10°59′00″ E	Tuscany	**S2**	Massiccio Del Matese	41°26′59.87″ N 14°22′19.21″ E	Molise/Campania
**C2**	Firenze Valdarno (FI)	43°39′24″ N 11°26′58″ E	Tuscany	**S3**	Campobasso (CB)	41°33′39.6″ N 14°40′06.24″ E	Molise
**C3**	Figline Valdarno (FI)	43°37′ N 11°28′ E	Tuscany	**S4**	Rodi Garganico (FG)	41°55′19.9″ N 15°52′37.86″ E	Puglia
**C4**	Grassina Ponte a Ema (FI)	43°44′22.42″ N 11°17′51.73″ E	Tuscany	**S5**	San Severo (FG)	41°41′42.4″ N 15°22′45.4″ E	Puglia
**C5**	Greve in Chianti (FI)	43°35′ N 11°19′ E	Tuscany	**S6**	San Basile (CS)	39°48′34.56″ N 16°09′47.81″ E	Calabria
**C6**	Reggello (FI)	43°41′ N 11°32′ E	Tuscany	**S7**	Cicala (CZ)	39°01′19.88″ N 16°29′09.96″ E	Calabria
**C7**	San Polo in Chianti (FI)	43°40′18.13″ N 11°21′46.13″ E	Tuscany	**I1**	Isola di Capraia (LI)	43°02′55.32″ N 9°50′25.08″ E	Tuscany
**C8**	Batignano (GR)	42°52′02.22″ N 11°09′57.84″ E	Tuscany	**I2**	Pianoconte (ME)	38°28′38.2″ N 14°55′43.8″ E	Sicily
**C9**	Montorsaio (GR)	42°53′26″ N 11°12′13″ E	Tuscany				

## Data Availability

Data are contained within the article and available on request.
